# Family-based case-control study of homotopic connectivity in first-episode, drug-naive schizophrenia at rest

**DOI:** 10.1038/srep43312

**Published:** 2017-03-03

**Authors:** Wenbin Guo, Feng Liu, Jindong Chen, Renrong Wu, Lehua Li, Zhikun Zhang, Jingping Zhao

**Affiliations:** 1 Department of Psychiatry of the Second Xiangya Hospital, Central South University, Changsha, Hunan 410011, China; 2 Key Laboratory for NeuroInformation of Ministry of Education, School of Life Science and Technology, University of Electronic Science and Technology of China, Chengdu, Sichuan, China; 3 Mental Health Center of the First Affiliated Hospital, Guangxi Medical University, Nanning, Guangxi, China

## Abstract

Family-based case-control design is rarely used but powerful to reduce the confounding effects of environmental factors on schizophrenia. Twenty-eight first-episode, drug-naive patients with schizophrenia, 28 family-based controls (FBC), and 40 healthy controls (HC) underwent resting-state functional MRI. Voxel-mirrored homotopic connectivity (VMHC), receiver operating characteristic curve (ROC), and support vector machine (SVM) were used to process the data. Compared with the FBC, the patients showed lower VMHC in the precuneus, fusiform gyrus/cerebellum lobule VI, and lingual gyrus/cerebellum lobule VI. The patients exhibited lower VMHC in the precuneus relative to the HC. ROC analysis exhibited that the VMHC values in these brain regions might not be ideal biomarkers to distinguish the patients from the FBC/HC. However, SVM analysis indicated that a combination of VMHC values in the precuneus and lingual gyrus/cerebellum lobule VI might be used as a potential biomarker to distinguish the patients from the FBC with a sensitivity of 96.43%, a specificity of 89.29%, and an accuracy of 92.86%. Results suggested that patients with schizophrenia have decreased homotopic connectivity in the motor and low level sensory processing regions. Neuroimaging studies can adopt family-based case-control design as a viable option to reduce the confounding effects of environmental factors on schizophrenia.

The “disconnection” hypothesis advanced that schizophrenia might be a result of aberrant brain connectivity^1^. Decreased functional connectivity (FC) has been reported in first-episode and chronic schizophrenia^2^,^3^. Decreased FC appears mainly among the frontal gyrus, temporal-parietal gyrus, anterior cingulate cortex, cerebellum, and corpus callosum^2^,^3^,^4^.

Decreased FC in schizophrenia involves disruptive integration of interhemispheric brain regions^2^,^5^. Interhemispheric interaction is mainly mediated by the corpus callosum, which links homotopic brain regions across the two hemispheres^6^. In healthy subjects, bihemispheric processing is advantageous in performing cognitive tasks^7^, provided that interhemispheric interaction is important in response to the given task. By contrast, patients with schizophrenia experience difficulty in bihemispheric processing. For example, neuroimaging studies revealed decreased callosal thickness in patients with schizophrenia^8^. The corpus callosum of patients with schizophrenia also exhibits reduced white matter density^9^,^10^. Furthermore, diffusion tensor imaging studies found decreased fractional anisotropy in the corpus callosum of patients with schizophrenia^11^, suggesting interhemispheric hypoconnectivity in patients with schizophrenia^12^. Using a voxel-mirrored homotopic connectivity (VMHC) measure^13^, two studies found reductions in FC between homotopic brain regions (mainly in brain regions of the default-mode network and the motor and low level sensory processing regions) in patients with schizophrenia^2^,^5^, provided that interhemispheric interaction deficits play a major role in the neurobiology of schizophrenia.

However, most of the above-mentioned studies used a traditional case-control design. Few studies employed a family-based case-control design, which is a powerful tool to limit the confounding effects of environmental risk factors on gene-related diseases, such as schizophrenia. The efficiency to identify genetic effects is acquired at the cost of potential overmatching on environmental risk factors^14^. Moreover, antipsychotic medication and long illness duration affect gray matter volume and brain connectivity in patients with schizophrenia^15^,^16^,^17^. This condition highlights the need to understand brain connectivity in first-episode, drug-naive patients with schizophrenia.

In this study, we applied a family-based case-control design to examine functional homotopy abnormalities in first-episode, drug-naive patients with schizophrenia. The first-episode, drug-naive patients were defined as having illness of less than 3 years, but never received antipsychotic treatment. The patients were matched with their unaffected siblings (family-based controls, FBC) at the family-based background. A group of healthy controls (HC) was also recruited in this study to compare the traditional case-control design and the family-based case-control design. Furthermore, only patients with paranoid schizophrenia were recruited to reduce disease heterogeneity, although the term “paranoid schizophrenia” disappeared in the new DSM-V system. VMHC was used to quantify the FC between the time series for a given voxel and that of its mirrored counterpart in the opposite hemisphere^13^. To date, this validated method has been applied in many psychiatric disorders, such as schizophrenia^2^,^5^ and unaffected siblings^18^, autism^19^, depression^20^,^21^, cocaine addiction^22^, and somatization disorder^23^. Given that decreased VMHC in brain regions of the default-mode network and the motor and low level sensory processing regions has been reported in patients with schizophrenia^2^,^5^, the present patients were expected to show decreased VMHC in certain brain regions, especially in brain regions of the default-mode network and the motor and low level sensory processing regions. The patients were also expected to show some correlations between decreased VMHC and symptom severity in consideration that such correlations have been observed in a previous study^2^.

## Materials and Methods

### Participants

This study was conducted in accordance with the Helsinki Declaration^24^. Thirty right-handed patients were recruited from Mental Health Center of the First Affiliated Hospital, Guangxi Medical University in China. Clinical diagnosis was made in accordance with the Structured Clinical Interview of the DSM-IV (SCID)^25^. To reduce the heterogeneity of symptom manifestations, only patients with paranoid schizophrenia were enrolled. The patients, who aged from 18 to 30 years old with more than 9 years of education, were first-episode and drug-naive at the scan time. The illness duration was less than 3 years. Patients who had neurological disorders, severe medical disorders, substance abuse, or electroconvulsive therapy were excluded. Symptom severity was rated using the Positive and Negative Symptom Scale (PANSS).

Thirty right-handed unaffected siblings of the patients were recruited as the FBC. Each patient was matched with one sibling from the same family. Forty-two right-handed HC were recruited from the community. The FBC and the HC were screened by SCID, non-patient edition^25^. The age of the controls ranged from 18 to 30 years, and their education level was more than 9 years. None of the controls had any neurological disorders, severe medical disorders, substance abuse or psychiatric disorders.

Written informed consent was acquired from each participant prior to data collection. This study was approved by the local ethics committee of the First Affiliated Hospital, Guangxi Medical University.

### Data acquisition and preprocessing

MRI images were obtained on a Siemens (Trio) 3T scanner. Data Processing Assistant for Resting-State fMRI (DPARSF) software (version 2.3)^26^ was applied to preprocess the images. Details on data acquisition and preprocessing were provided in Supplementary files.

### VMHC analysis

VMHC analysis was conducted with the REST software (version 1.8)^27^. For each participant, homotopic FC was calculated as the Pearson correlation coefficient between each voxel’s residual time series and that of its interhemispheric mirrored voxel. The coefficients were then Fisher *z*-transformed, and VMHC maps were generated for each participant.

For each group, one-sample *t*-tests were applied to identify voxels that exhibited significant correlations with their mirrored counterparts. The significance level was set at *p* < 0.05 for multiple comparisons corrected by Gaussian Random Field (GRF) theory (voxel significance: *p* < 0.001, cluster significance: *p* < 0.05). Analyses of covariance (ANCOVA), followed by post hoc *t*-tests (paired-sample *t*-tests were used to compare group differences between the patients and the FBC), were performed to compare group differences within the union mask of one-sample *t*-tests of the three groups. Age and years of education were used as covariates to minimize the potential effects of these variables. Framewise displacement (FD) values were computed for each participant as described in a previous study^28^, and FD was used as a covariate in the group comparisons. The significance level was set at *p* < 0.05 (GRF corrected).

### Correlation analysis

Once significant group differences were found in any brain regions, the mean *z* values of these brain regions were extracted to assess the correlations between abnormal VMHC values and symptom severity in the patients. The correlation results were Bonferroni corrected at *p* < 0.05.

To determine whether abnormal VMHC values were correlated in the patients and the FBC, we made a mask on the deficit clusters of the VMHC results between the patients and the FBC. Then, the mean *z* values within the mask were extracted in the patients and the FBC. Finally, Pearson correlation analysis was performed on the abnormal VMHC values between the patients and the FBC. In addition, the correlations between abnormal VMHC values for each cluster were computed between the patients and the FBC.

### Receiver operating characteristic curve (ROC) analysis and support vector machine (SVM) analysis using LIBSVM

ROC analysis was conducted to determine whether VMHC in the brain regions with group differences can differentiate the patients from the controls.

The LIBSVM software package (http://www.csie.ntu.edu.tw/~cjlin/libsvm/)^29^ in Matlab was applied to explore the possibility of combining these clusters to distinguish the patients from the HC/FBC. The Grid search method and Gaussian radial basis function kernels were used for parameter optimization. The “leave-one-out” cross-validation method was applied to obtain the highest sensitivity and specificity^30^,^31^,^32^.

## Results

### Demographic and clinical characteristics

One patient, one FBC, and two HC who had excessive head movement were excluded from the analysis. Hence, 28 patient-FBC pairs and 40 HC were enrolled in the final analysis. As listed in Table 1, the three groups exhibited no significant differences in age, sex ratio, years of education, and FD values. The illness duration for the patients was approximately 24 months. The patients obtained a mean PANSS total score of 88.

### VMHC patterns

For each group, brain voxels exhibited homotopic connectivity with their mirrored counterparts (Figures S1–S3). A union mask was generated from the results of one-sample *t*-tests for the following VMHC analysis.

### Group differences in VMHC

**Figure 1:**
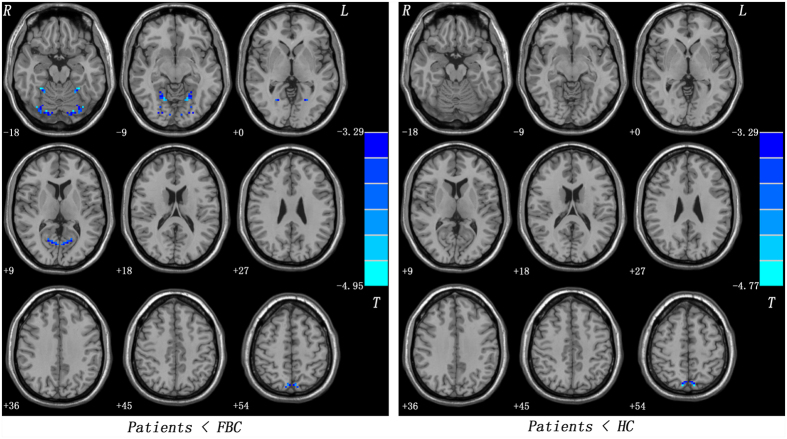
Statistical maps showing decreased VMHC in the patients compared to the FBC/HC. Blue denotes lower VMHC in the patients and the color bars indicate the *T* value from post hoc *t*-tests. VMHC = voxel-mirrored homotopic connectivity; FBC = family-based controls; HC = healthy controls.

As shown in Figure S4, ANCOVA revealed significant group differences in VMHC among the cerebellum, precuneus, fusiform gyrus, and lingual gyrus. Compared with the FBC, the patients showed decreased VMHC in the precuneus, fusiform gyrus/cerebellum lobule VI, and lingual gyrus/cerebellum lobule VI (Fig. 1 and Table 2). The patients exhibited lower VMHC in the precuneus relative to the HC (Fig. 1 and Table 2). No regions with abnormal VMHC were observed between the FBC and the HC.

### Correlations, ROC and SVM results

**Figure 2:**
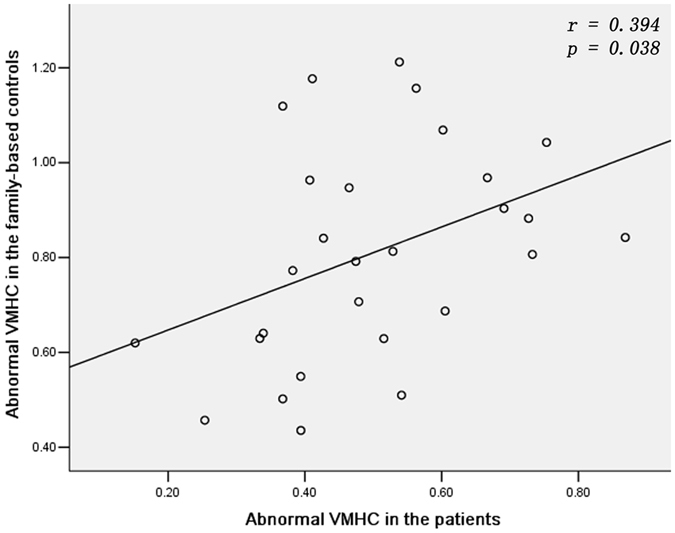
A positive correlation between the abnormal VMHC values across all three deficit clusters in the patients and the FBC. VMHC = voxel-mirrored homotopic connectivity, FBC = family-based controls.

No correlations were found between decreased VMHC and symptom severity (PANSS scores) in the patients. In addition, decreased VMHC showed no correlation with age and years of education in the patients. A positive correlation was observed between the abnormal VMHC values across all three deficit clusters in the patients and the FBC (*r* = 0.394, *p* = 0.038, Fig. 2). For each cluster, a positive correlation was observed between the abnormal VMHC values of the fusiform gyrus/cerebellum lobule VI in the patients and the FBC (*r* = 0.439, *p* = 0.019, Figure S5), and no correlations were found between abnormal VMHC values of the other two clusters in the patients and the FBC (lingual gyrus/cerebellum lobule VI: *r* = 0.349, *p* = 0.068; precuneus: *r* = 0.147, *p* = 0.456, Figure S5).

**Figure 3:**
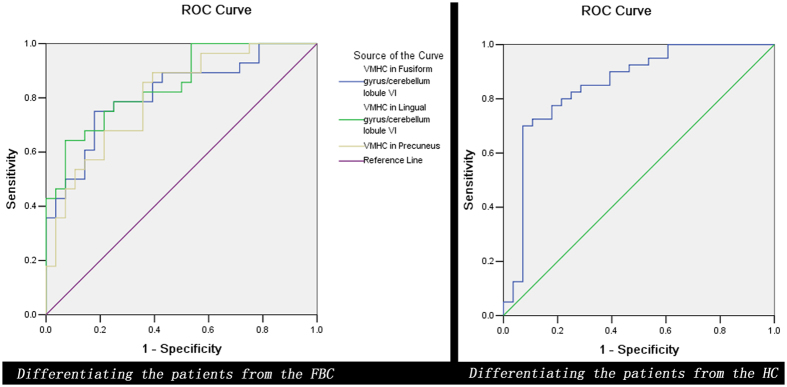
Receiver operating characteristic (ROC) curves using the mean VMHC values in brain regions with group differences to separate the patients from the controls. FBC = family-based controls; HC = healthy controls; VMHC = voxel-mirrored homotopic connectivity.

**Figure 4:**
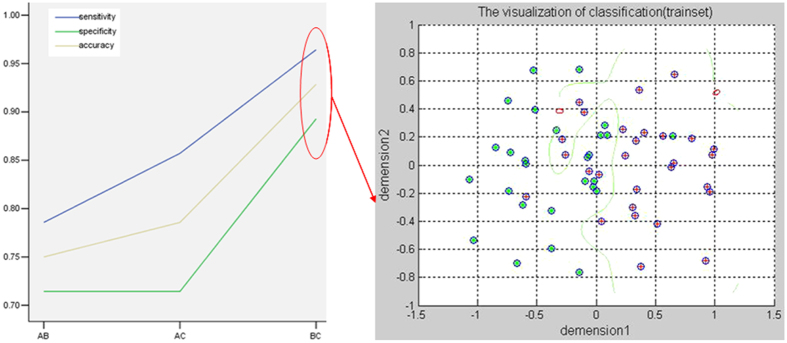
Performance for VMHC-based SVM classification to separate the patients from the FBC. Sensitivity, specificity, and accuracy curves for combination of VMHC values in the three clusters were labeled with different colors. **A**,**B** and **C** represent fusiform gyrus/cerebellum lobule VI, lingual gyrus/cerebellum lobule VI, and precuneus, respectively. VMHC = voxel-mirrored homotopic connectivity, SVM = support vector machine, FBC = family-based controls.

Further ROC analysis exhibited that none of the four clusters with group differences can be used to differentiate the patients from the HC/FBC with satisfactory sensitivity and specificity (Fig. 3 and Table 3). Thus, SVM analysis was conducted to determine whether combining of the VMHC values in these brain regions can distinguish the patients from the FBC with optimal sensitivity and specificity. Results showed that 52 subjects were correctly classified after combining the VMHC values in the precuneus and lingual gyrus/cerebellum lobule VI with a sensitivity of 96.43%, a specificity of 89.29%, and an accuracy of 92.86%, which was the optimal combination with the highest accuracy (Fig. 4).

## Discussion

Using a family-based case-control design, the present study reveals decreased homotopic connectivity in the motor and low level sensory processing regions (the precuneus, fusiform gyrus/cerebellum lobule VI, and lingual gyrus/cerebellum lobule VI) of first-episode, drug-naive patients with schizophrenia. No correlations can be observed between decreased VMHC and clinical symptoms (PANSS scores).

The most novel aspect of this study is the application of a family-based case-control design. Current understanding of schizophrenia is that this disease may be the result of interactions between several genes with minimal effects and environmental factors^33^. The traditional case-control design in schizophrenia is a powerful tool to detect group differences between the patients and the controls. However, the traditional case-control design may be confounded by environmental factors even though the controls are from the same large geographic area as the patients. Group differences revealed by the traditional case-control design may have been biased by unmatched effects of environmental factors. In the present study, the traditional case-control design reveals that only the precuneus shows decreased VMHC in the patients relative to the HC. By contrast, the family-based case-control design reveals that the patients have decreased VMHC in the fusiform gyrus/cerebellum lobule VI and lingual gyrus/cerebellum lobule VI in addition to the precuneus compared with the FBC. Therefore, the family-based case-control design minimizes bias due to unmatched cases and controls because the patient-FBC pairs are well matched on many genetic and ethnic confounders. This condition enhances the specificity of detecting possible biomarkers for schizophrenia. However, the efficiency of the family-based case-control design is obtained at the cost of potential overmatching on environmental risk factors^14^. This issue is partly supported by the positive correlations between abnormal VMHC values within the deficit clusters (especially the fusiform gyrus/cerebellum lobule VI) in the patients and the FBC. The shared genetic and overmatched environmental factors may contribute to the positive correlations between abnormal VMHC values within the deficit clusters in the patients and the FBC.

Using the family-based case-control design, we replicated part of our previous findings^2^ (i.e., reduced VMHC in the precuneus, fusiform gyrus/cerebellum lobule VI, and lingual gyrus/cerebellum lobule VI). The precuneus includes several functional subregions^34^, and the deficit cluster in the present study locates at the dorsal-posterior precuneus that is involved in visual-spatial information processing^35^. The fusiform gyrus participates in face and body recognition^36^. The lingual gyrus is associated with processing vision, especially associated with letters^37^. The cerebellum is involved in motor coordination, precision, and accurate timing^38^. Our findings are consistent with a previous study that reported reduced VMHC in the cerebellum and posterior low level sensory processing areas in patients with schizophrenia and schizoaffective disorder^5^. Replicated findings suggest that reduced VMHC in the four regions is special to patients with schizophrenia because the family-based case-control design is applied in the present study to reduce the confounding effect of environmental factors. Reduced VMHC in the four brain regions indicates that these regions have a problem in homotopic coordination, which further affects the function of the motor and low level sensory processing circuits in the patients.

Correlations between VMHC in certain brain regions and PANSS scores have been reported in previous studies^2^,^39^, implicating that VMHC reductions bear clinical significance in psychiatric symptomatology. However, this study reveals no correlations between decreased VMHC and PANSS scores. Two possibilities exist for the inconsistency. First, the present findings revealed by the family-based case-control design may be trait alterations independent of symptom severity. VMHC in the precentral gyrus and superior temporal gyrus that showed correlations with PANSS scores in the previous study^2^ does not exhibit group differences in the present study. The absence of VMHC findings in the two regions may have caused the lack of correlations between VMHC and PANSS scores in the present study. Second, predominantly positive and negative symptoms in the present patients may complicate the correlations between VMHC and clinical symptoms. For example, Cho *et al*.^40^ reported a significantly positive correlation between thalamo-orbitofrontal cortex connectivities and Global Assessment of Functioning scores in subjects with ultra-high risk for psychosis. However, this correlation disappeared in first-episode patients with schizophrenia.

An ideal biomarker allows discrimination between the patients and the HC with high sensitivity/specificity of more than 75%^41^. For this criterion, ROC analysis exhibits that the VMHC values in these brain regions may not be ideal biomarkers to separate the patients from the HC/FBC, suggesting that the discriminative power from a single cluster is limited to differentiate the patients from the HC/FBC. Further SVM analysis indicates that a combination of VMHC values in the precuneus and lingual gyrus/cerebellum lobule VI may be used as a potential biomarker to separate the patients from the FBC with a sensitivity of 96.43%, a specificity of 89.29%, and an accuracy of 92.86%. Regrettably, only one cluster exhibits significant differences between the patients and the HC. This issue prevents us to differentiate the patients from the HC by using a combination of VMHC values in two or more clusters with the SVM analysis though it is important to do so.

In addition to the relatively small sample size, several limitations should be addressed. First, previous studies^42^,^43^,^44^,^45^,^46^,^47^ reported that the patients and the FBC share abnormal brain activity and connectivity in certain regions, which may be trait alterations for schizophrenia. When the family-based case-control design is used, the effects of shared abnormal brain activity and connectivity may be over-matched by the design, and therefore are excluded from the final results. However, no significant differences in VMHC values can be observed between the FBC and the HC in this study. This condition suggests that abnormal VMHC in certain brain regions shared by the patients and the FBC is subtle. Thus, the effects of the shared abnormalities between the patients and the FBC exert minimal influence on the final results. Second, the group differences in years of education show a trend level of significance. The patients received fewer years of education than the controls. Although this variable was applied as a covariate in the analysis, the effect of unmatched education level may not be eliminated completely in this study. Third, the brain cortex is asymmetrical, and the usage of a symmetrical standard template in the analysis may have biased the present findings. Fourth, the inhomogeneous B0 field usually induced distortion in brain regions such as the prefrontal cortex and the cerebellum. Thus, the present findings should be explained with caution. Future studies should be designed to correct such distortion. Finally, this study is cross-sectional; longitudinal studies are needed to clarify the changes in VMHC after antipsychotic treatment.

Despite the limitations, the family-based case-control design used in the present study indicates that patients with schizophrenia exhibit decreased homotopic connectivity in the motor and low level sensory processing regions. The present findings suggest evidence of homotopic disconnection in patients with schizophrenia. The family-based case-control design can serve as a viable option in neuroimaging studies to reduce the confounding effects of environmental factors on complex diseases, such as schizophrenia.

## Additional Information

**How to cite this article**: Guo, W. *et al*. Family-based case-control study of homotopic connectivity in first-episode, drug-naive schizophrenia at rest. *Sci. Rep.* **7**, 43312; doi: 10.1038/srep43312 (2017).

**Publisher's note:** Springer Nature remains neutral with regard to jurisdictional claims in published maps and institutional affiliations.

## Supplementary Material

Supplementary Files

## Figures and Tables

**Table 1:** Demographic and clinical characteristics of the participants.

	Patients (n = 28)	FBC (n = 28)	HC (n = 40)	*P* value
Sex (male/female)	18/10	21/7	20/20	0.106^a^
Age (years)	22.93 ± 3.92	22.86 ± 3.14	23.28 ± 2.60	0.843^b^
Years of education (years)	10.54 ± 2.32	11.93 ± 2.54	11.53 ± 1.81	0.053^b^
FD (mm)	0.05 ± 0.04	0.05 ± 0.02	0.06 ± 0.03	0.942^b^
Illness duration (months)	24.14 ± 7.17			
PANSS				
Positive scores	22.68 ± 5.64			
Negative scores	21.18 ± 5.40			
Total scores	88.11 ± 10.29			

^a^The *P* value for sex distribution was obtained by chi-square test.

^b^The *P* values were obtained by analysis of variance.

FBC = family-based controls; HC = healthy controls; FD = framewise displacement; PANSS = Positive and Negative Symptom Scale.

**Table 2:** Regions with increased VMHC in the patients.

Cluster location	Peak (MNI)			Number of voxels	*T* value
	x	y	z		
*Patients* < *FBC*					
Precuneus	±6	−75	51	26	−4.6477
Fusiform gyrus/cerebellum lobule VI	±27	−45	−15	120	−4.8343
Lingual gyrus/cerebellum lobule VI	±21	−81	−18	108	−4.8707
*Patients* < *HC*					
Precuneus	±6	−63	69	64	−4.7738
*FBC vs HC*					
None					

FBC = family-based controls; HC = healthy controls; MNI = Montreal Neurological Institute; VMHC = voxel-mirrored homotopic connectivity.

**Table 3:** ROC analysis for differentiating the patients from the controls.

Brain regions	Area Under the Curve	Cut-off point	Sensitivity	Specificity
*Differentiating the patients from the FBC*				
Precuneus	0.805	0.8236^a^	64.29% (18/28)	85.71% (24/28)
Fusiform gyrus/cerebellum lobule VI	0.818	0.5866	82.14% (23/28)	75.00% (21/28)
Lingual gyrus/cerebellum lobule VI	0.849	0.6917	92.86% (26/28)	64.29% (18/28)
*Differentiating the patients from the HC*				
Precuneus	0.851	0.9241	92.86% (26/28)	70.00% (28/40)

^a^By this cut-off point, the VMHC value of the precuneus could correctly classify 18 of 28 patients and 24 of 28 FBC, resulted in a sensitivity of 64.29% and a specificity of 85.71%. The meanings of other cut-off points were similar.

ROC = receiver operating characteristic curves; FBC = family-based controls; HC = healthy controls; VMHC = voxel-mirrored homotopic connectivity.
