# Supplementary material for: Family-based case-control study of homotopic connectivity in first-episode, drug-naive schizophrenia at rest
Source: Sci Rep. 2017 Mar 3;7:43312. doi: 10.1038/srep43312 (PMC5335664; doi:10.1038/srep43312)
Supplement: Supplementary Files [file srep43312-s1.pdf]

Title:

Family-based case-control study of homotopic connectivity in first-episode, drug-naive schizophrenia at rest

Authors:

Wenbin Guo[1] Feng Liu[2] Jindong Chen[1] Renrong Wu[1] Lehua Li[1] Zhikun Zhang[3] Jingping Zhao[1]

Affiliation/address:

1. Department of Psychiatry of the Second Xiangya Hospital, Central South University, Changsha, Hunan 410011, China.
2. Key Laboratory for NeuroInformation of Ministry of Education, School of Life Science and Technology, University of Electronic Science and Technology of China, Chengdu, Sichuan, China.
3. Mental Health Center of the First Affiliated Hospital, Guangxi Medical University, Nanning, Guangxi, China

# Corresponding authors:

Wenbin Guo
Department of Psychiatry of the Second Xiangya Hospital, Central South University, Changsha, Hunan 410011, China.
E-mail: guowenbin76@163.com
Tel.: +86 731 85360921

Jingping Zhao
Department of Psychiatry of the Second Xiangya Hospital, Central South University, Changsha, Hunan 410011, China.
E-mail: zhaojingpingcsu@163.com
Tel.: +86 731 85360921

Data acquisition and preprocessing

MRI images were obtained on a Siemens (Trio) 3T scanner. The participants were required to remain motionless and awake with their eyes closed. Soft earplugs and foam pads were used to decrease scanner noise and head motion. Resting-state functional images were obtained with a gradient-echo echo-planar imaging (EPI) sequence using the following parameters: repetition time/echo time = 2000 ms/30 ms, 30 slices, 64 × 64 matrix, 90° flip angle, 240 mm field of view, 4 mm slice thickness, 0.4 mm gap, and 250 volumes lasting for 500 s. High-resolution T1-weighted images were also obtained with a three-dimensional spoiled gradient-recalled sequence in an axial orientation: repetition time = 8.5 ms, echo time = 2.98 ms, inversion time = 900 ms, flip angle = 9°, acquisition matrix = 256 × 256, field of view = 240 mm × 240 mm, slice thickness = 1 mm, no gap, and 176 slices. After the scan, each participant was asked some questions to confirm the wakefulness during the scan.

Data Processing Assistant for Resting-State fMRI (DPARSF) software (version 2.3) [1] was applied to preprocess the images. After slice timing and head motion correction, participants with more than 2 mm of maximal translation and 2° of maximal rotation were excluded. Then, we performed the following steps [2]. First, the T1 images were reoriented and coregistered to the mean functional images. Subsequently, the T1 images were segmented into gray matter, white matter, and cerebrospinal fluid by using a unified segmentation algorithm [3]. Next, the motion-corrected functional volumes were spatially normalized to the MNI space and resampled to $3 \times 3 \times 3$ mm^3 voxels using the normalization parameters estimated during unified segmentation. After normalization, the images were smoothed (with an 8 mm full width at half maximum Gaussian kernel), bandpass filtered (0.01 - 0.08 Hz), and linearly detrended. Several covariates, including

Friston-24 head motion parameters acquired by rigid body correction, signal from a ventricular region of interest (ROI), and signal from a region centered in the white matter, were removed. The global signal was not removed as indicated in a previous study [4].

References

1 Yan, C. & Zang, Y. DPARSF: A MATLAB toolbox for "pipeline" data analysis of resting-state fMRI. *Front Syst Neurosci* **4**, 13 (2010).

2 Kybic, J., Thevenaz, P., Nirkko, A. & Unser, M. Unwarping of unidirectionally distorted EPI images. *IEEE Trans Med Imaging* **19**, 80-93 (2000).

3 Ashburner, J. & Friston, K. J. Unified segmentation. *Neuroimage* **26**, 839-851 (2005).

4 Hahamy, A. *et al.* Save the global: global signal connectivity as a tool for studying clinical populations with functional magnetic resonance imaging. *Brain Connect* **4**, 395-403 (2014).

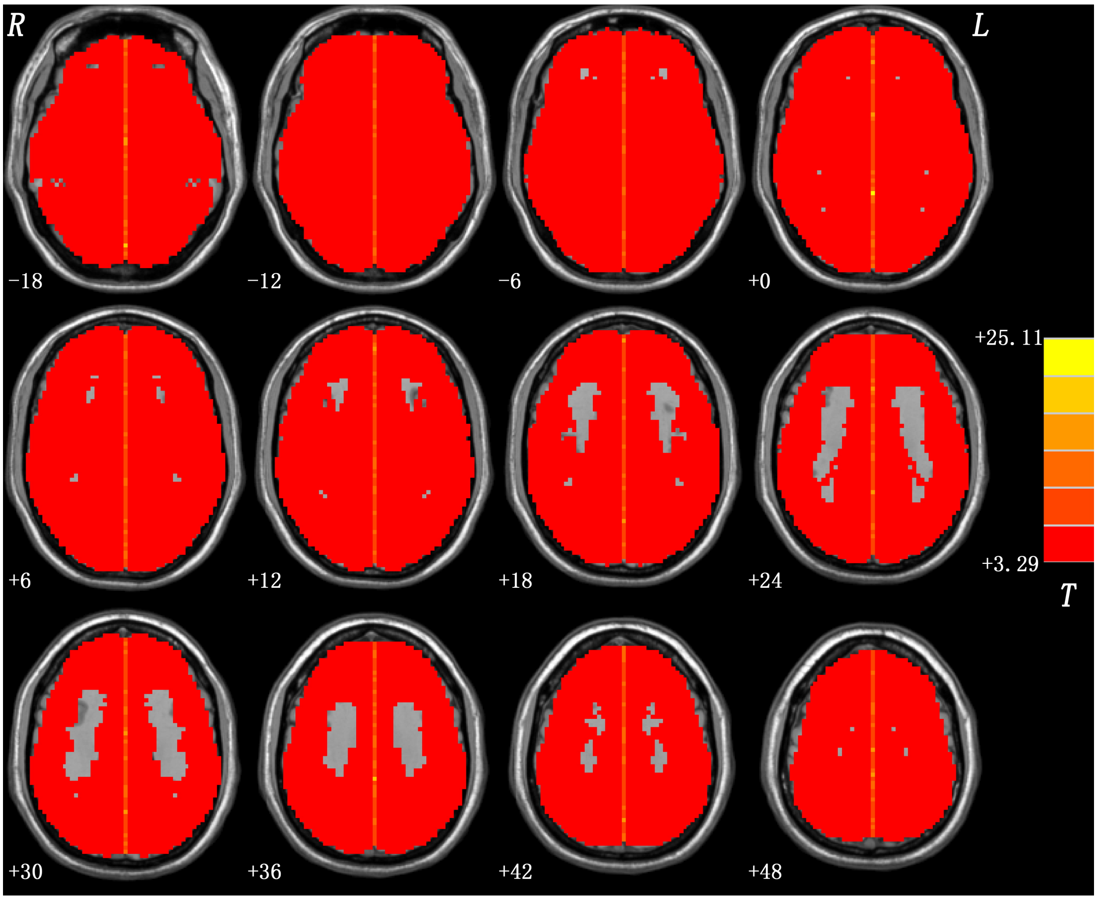

Figure S1. Statistical maps showing brain voxels that exhibited homotopic connectivity with their mirrored counterparts in patients with schizophrenia. Color bars indicate the *T* value from

one-sample *t*-tests between groups. VMHC = voxel-mirrored homotopic connectivity.

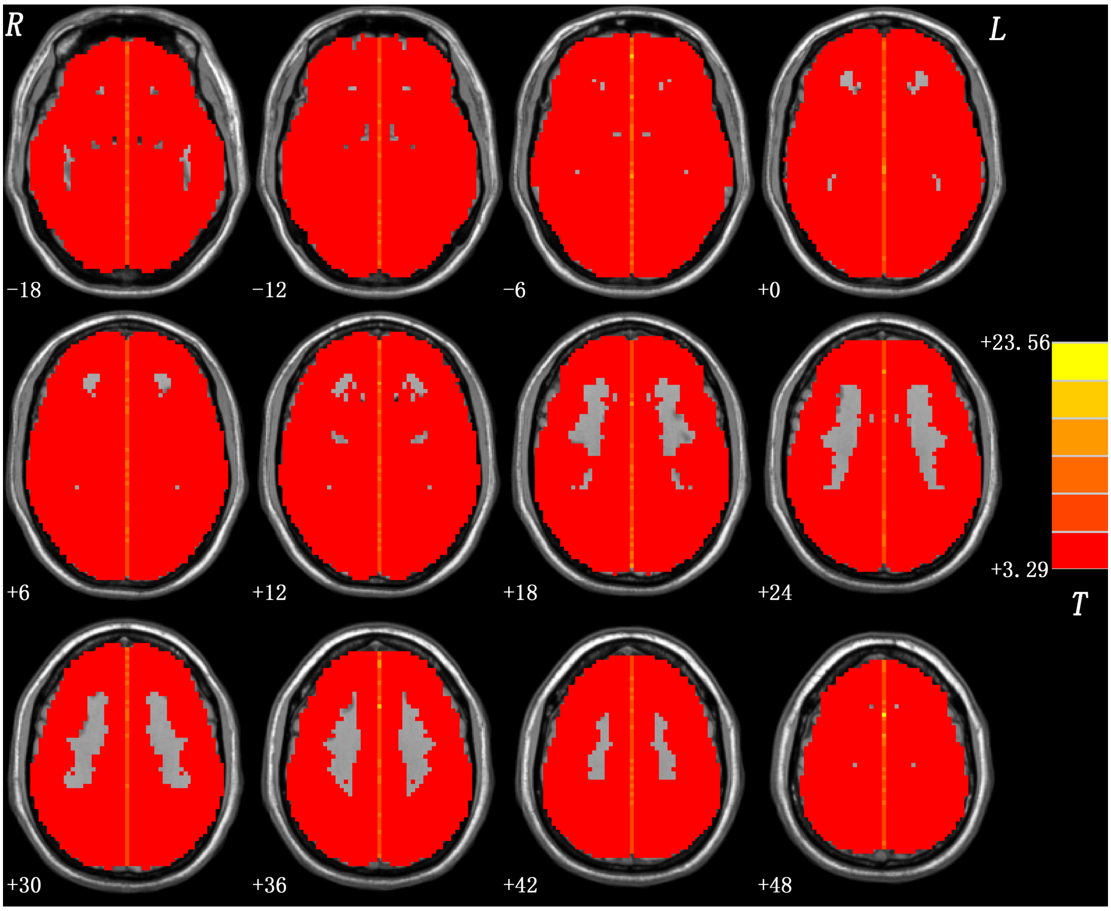

Figure S2. Statistical maps showing brain voxels that exhibited homotopic connectivity with their mirrored counterparts in the unaffected siblings (family-based controls). Color bars indicate the *T* value from one-sample *t*-tests between groups. VMHC = voxel-mirrored homotopic connectivity.

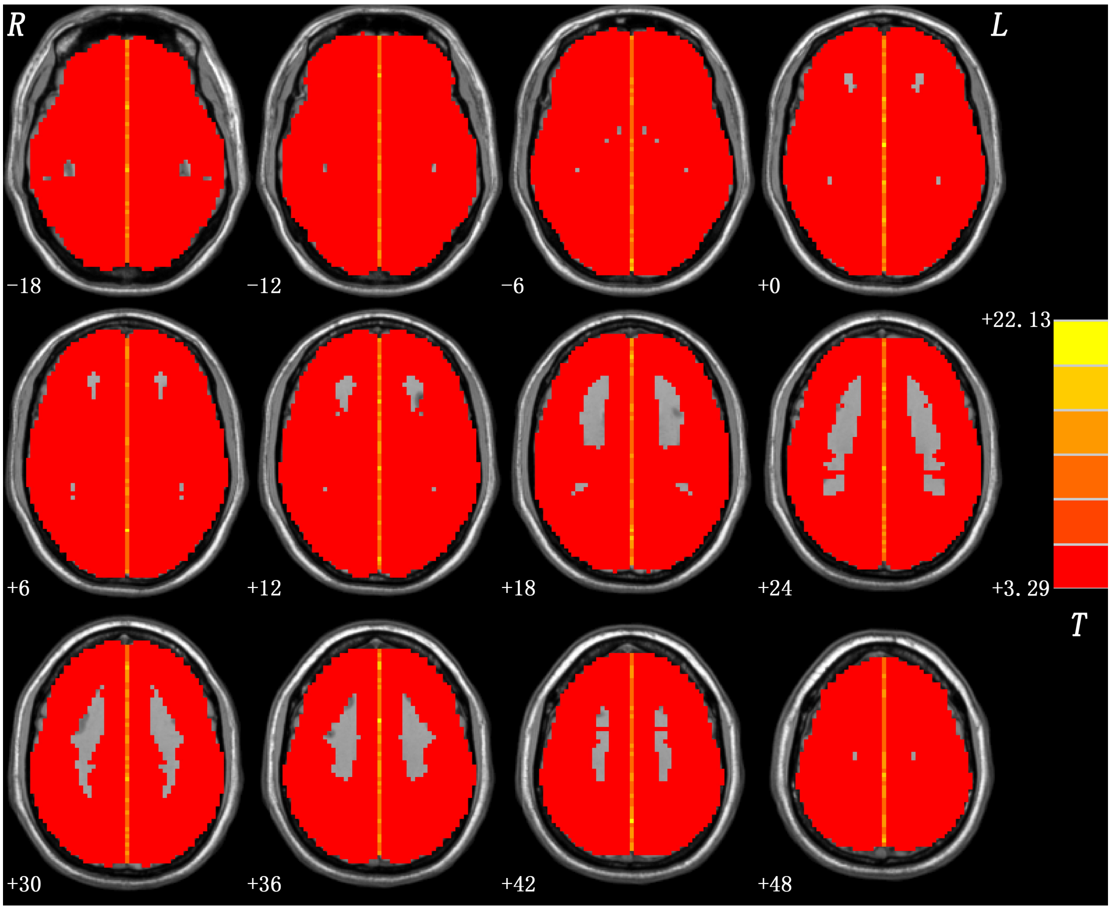

Figure S3. Statistical maps showing brain voxels that exhibited homotopic connectivity with their mirrored counterparts in the healthy controls. Color bars indicate the *T* value from one-sample *t*-tests between groups. VMHC = voxel-mirrored homotopic connectivity.

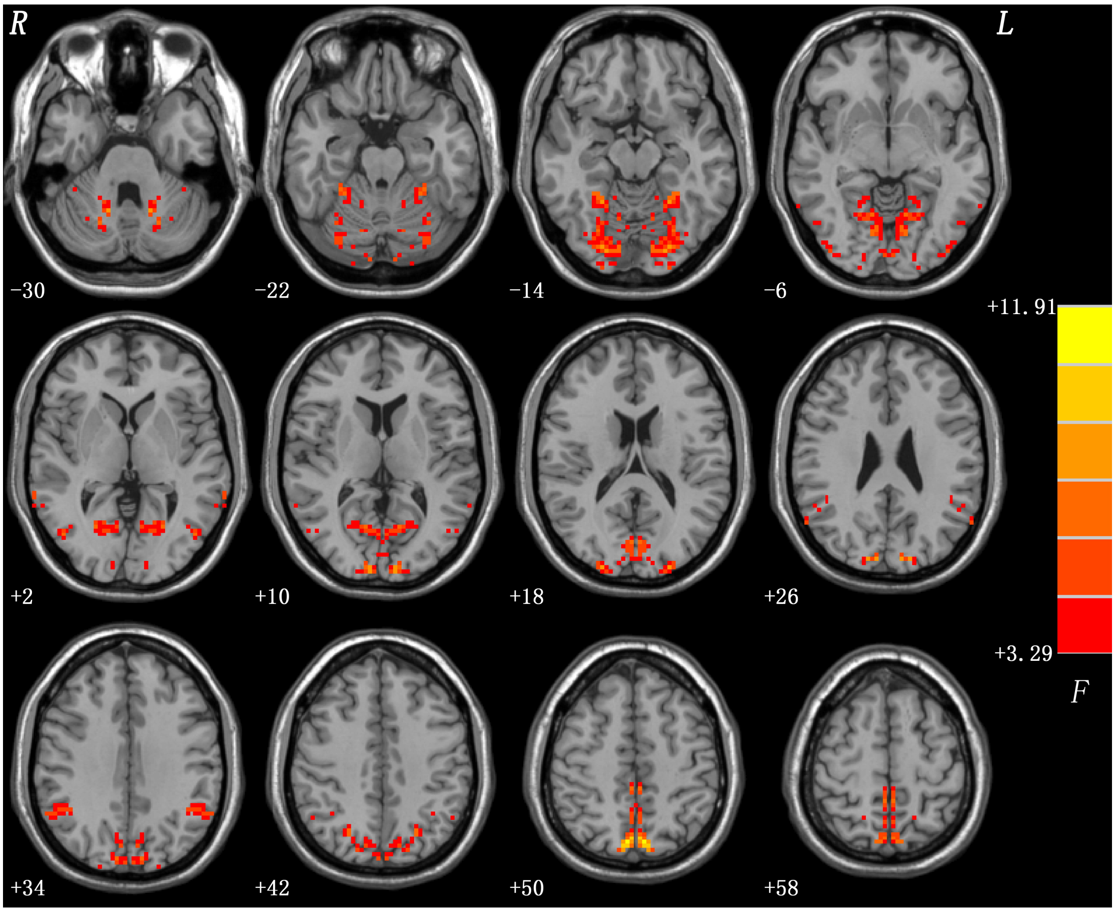

Figure S4. Statistical maps showing significant group differences in VMHC revealed by ANCOVA. Color bars indicate the *F* value from ANCOVA. VMHC = voxel-mirrored homotopic connectivity, ANCOVA = analyses of covariance.

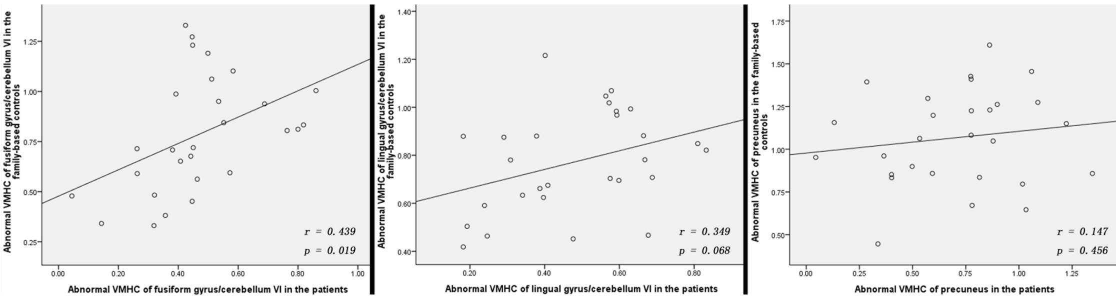

Figure S5. A positive correlation between the abnormal VMHC values of the fusiform gyrus/cerebellum lobule VI in the patients and the FBC, and no correlations between abnormal VMHC values of the other two clusters (the lingual gyrus/cerebellum lobule VI and the precuneus)

in the patients and the FBC. VMHC = voxel-mirrored homotopic connectivity, FBC = family-based controls.
